# Supplementary material for: IbMYB308, a Sweet Potato R2R3-MYB Gene, Improves Salt Stress Tolerance in Transgenic Tobacco
Source: Genes (Basel). 2022 Aug 18;13(8):1476. doi: 10.3390/genes13081476 (PMC9408268; doi:10.3390/genes13081476)
Supplement: Supplementary file 1 [file genes-13-01476-s001.zip › FigureS2.pdf]

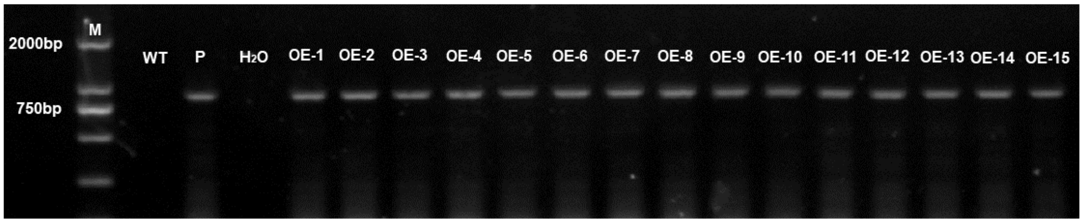

Figure S2. PCR Detection of *IbMYB308* overexpression tobacco. OE-1-15: *IbMYB308* transgenictobacco; WT: wild-typetobacco; P: overexpression plasmid of *IbMYB308*. M: DNA Maker DL2000.
