# Supplementary material for: IbMYB308, a Sweet Potato R2R3-MYB Gene, Improves Salt Stress Tolerance in Transgenic Tobacco
Source: Genes (Basel). 2022 Aug 18;13(8):1476. doi: 10.3390/genes13081476 (PMC9408268; doi:10.3390/genes13081476)
Supplement: Supplementary file 1 [file genes-13-01476-s001.zip › TableS1.pdf]

Table S1. Primers used in this experiment

Primer name	Primer sequences (5′-3′)
IbMYB308-F	ATGGGAAGGTCTCCTTGCT
IbMYB308-R	CTAATTGCTGCTGCTGCTG
*IbMYB308*pro-F	ACACACCCATTTTCGTCCTT
*IbMYB308*pro-R	CTTTTTCGCAGCAAGGAGAC
IbMYB308-F1	GCCCACCGTCGTCCCAAGAAA
IbMYB308-R1	AAGCACAACCCGTTGACCCTC
IbMYB308-F2	GCGCGGTACCATGGGAAGGTCTCCTTGCT
IbMYB308-R2	GCGCGGATCCCTAATTGCTGCTGCTGCTG
IbActin-F	AGCAGCATGAAGATTAAGGTTGTAGCAC
IbActin-R	TGGAAAATTAGAAGCACTTCCTGTGAAC
35S-F	TTGATGTGATATCTCCACTGACG
1300-R	GCGCGGATCCCTAATTGCTGCTGCTGCTG
NtActin-F	GAGGAATGCAGATCTTCGTG
NtActin-R	TCCTTGTCCTGGATCTTAGC
SOD-F	CTATTACCGACAAGCAGATTCCTC
SOD-R	TACCACAAGCAACCCTTCCAC
POD-F	TCCGGGAGCCACACCATTGG
POD-R	TGGTCGGAATTCAACAG
APX-F	GATGTTCCCTTTCACCCTGG
APX-R	CAGATAGACCCATTTGCTTCACA
P5CS-F	TTGTGACACGGACTGATGGAA
P5CS-R	TATCTAAGCCGCTGACGACCA
