# Supplementary figures and images for: IbMYB308, a Sweet Potato R2R3-MYB Gene, Improves Salt Stress Tolerance in Transgenic Tobacco
Source: Genes (Basel). 2022 Aug 18;13(8):1476. doi: 10.3390/genes13081476 (PMC9408268; doi:10.3390/genes13081476)

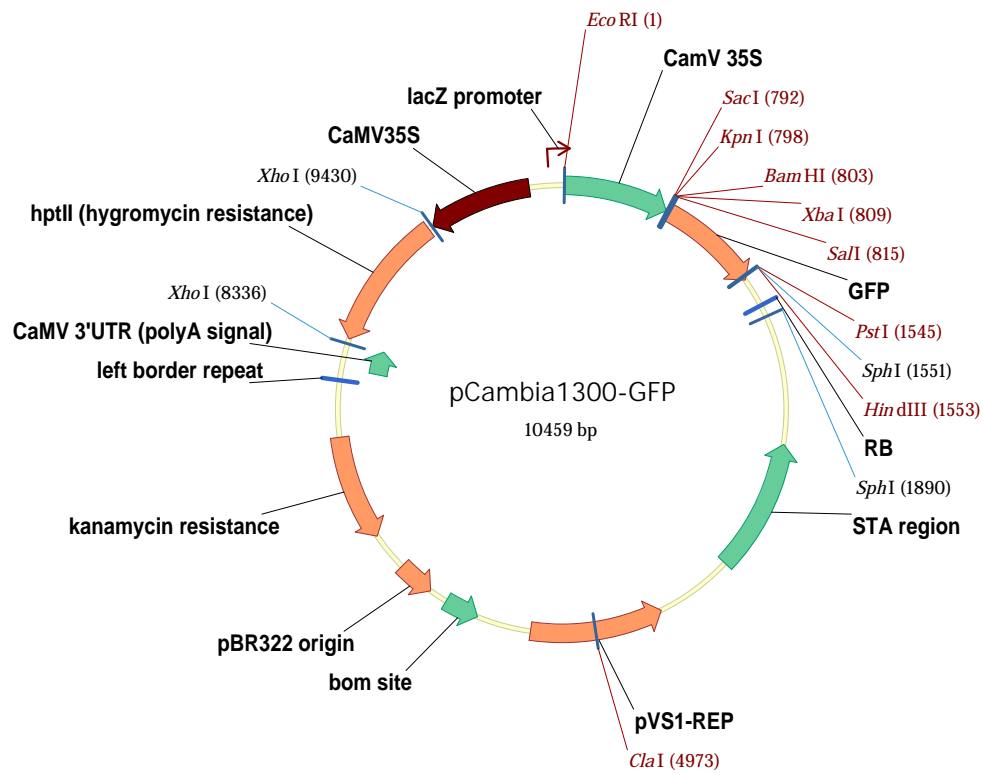

(a)

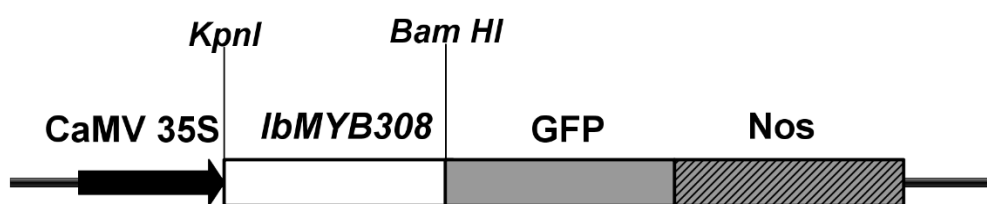

(b)

**Figure S1.** (a) pCAMBIA1300 vector, (b) Recombinant plasmid pCAMBIA-IbMYB308-GFP

Supplement: Supplementary file 1 [file genes-13-01476-s001.zip › FigureS1.pdf]
